# Supplementary material for: Human Sperm Morphology as a Marker of Its Nuclear Quality and Epigenetic Pattern
Source: Cells. 2022 May 30;11(11):1788. doi: 10.3390/cells11111788 (PMC9180039; doi:10.3390/cells11111788)
Supplement: Supplementary file 1 [file cells-11-01788-s001.zip › cells-1727561-supplementary.pdf]

**Table S1.** Compliance with the checklist for acceptability of studies based on human semen analysis, by Björndahl et al., 2015 [48].

**Patients**

☒ For clinical studies: The patient population (e.g., patients, volunteers, students) has been declared in the manuscript, together with the recruitment method and inclusion and exclusion criteria. If the study concerns couples being investigated for infertility, then the following must be specified in the manuscript: fertility status of female partner; and primary, secondary or other level of investigation of the man.

NB: The sperm characteristics presented in this study were used only to classify patients, and they were not used here as variables affecting our results.

☐ If used in the manuscript, the term "male factor" must be completely defined.

NB: Not applicable. This term is not used in the manuscript.

**General aspects**

☒ Patients were instructed to maintain 2–7 days of sexual abstinence before collecting a sample for investigation.

☒ Patients were informed about the importance of reporting any missed early ejaculate fractions, and men's answers were noted on the laboratory form.

☐ For specimens not collected at the laboratory, patients were instructed to avoid cooling or heating of the semen sample during transport to the laboratory.

NB: Not applicable. All patients performed semen collection in the laboratory.

☒ Samples were kept at 37°C before initiation of and during the analysis in case of sperm motility assessment.

☒ For samples collected adjacent to the laboratory, analysis was initiated after completion of liquefaction and within 30 min after ejaculation. If this was not done—and more importantly when some of the samples are collected in the laboratory and others are collected at home—it should be checked that this did not influence the data (and, if yes, that this effect must be included as a confounding factor in the statistical analysis).

☒ Liquefaction was first checked within 30 min after ejaculation. A Volume was determined either by weighing or using a wide-bore volumetric pipette.

☒ Viscosity was measured using either a wide-bore pipette or a glass rod.

☒ All staff members who performed the analyses have been trained in basic semen analysis (ESHRE Basic Semen Analysis Course—or equivalent—and further in-house training) and participate regularly in internal quality control.

☒ If more than one method can be recommended for a particular characteristic (e.g., to measure volume), only one should be used in a given study.

**Sperm concentration assessment**

☒ Semen aliquot to be diluted for sperm concentration assessment was taken with a positive displacement pipette (i.e., a 'PCR pipette') using a recommended diluent (state which diluent: Formol saline).

☒ Only standard dilutions were used (1:50, 1:20, or 1:10).

☒ Sperm concentration was assessed using haemocytometers with improved Neubauer ruling.

☒ Haemocytometers were allowed to rest for 10–15 min in a humid chamber to allow sedimentation of the suspended spermatozoa onto the counting grid before counting.

☒ Sperm counting was done using phase contrast microscope optics (200–400×).

☒ Comparisons were made between duplicate counts, and counts re-done when the difference exceeded the acceptance limits.

☒ Typically at least 200 spermatozoa were counted in each of the duplicate assessments.

**Sperm motility assessment**

☒ Motility assessments were performed at 37°C ± 0.5°C.

☒ Motility assessments were done using phase contrast microscope optics (200–400×).

☒ Sperm motility was classified using a four-category scheme: rapid progressive, slow progressive, non-progressive, and immotile (World Health Organization, 1999; Björndahl et al., 2010; Barratt et al., 2011).
☒ Motility assessments were done in duplicate and compared; counts were re-done on new preparations when the difference between duplicates exceeded the acceptance limits.
☒ The wet preparation was made with a drop of 10 μL and a 22x 22 mm coverslip to give a depth of 20 mm (must be at least 10 mm, but not too deep so as to allow spermatozoa to move freely in and out of focus; typically ca. 20 mm).
☒ At least 200 spermatozoa were assessed in each duplicate motility count.
☒ At least 5 microscope fields of view were examined in each duplicate count.

**Sperm vitality assessment**
☒ A validated supravital staining, appropriate to the type of microscope optics utilized, was used to assess sperm vitality.
☒ At least 200 spermatozoa were evaluated in each sample.

☒ Assessments were done under high magnification (×1000– 1250) using a 100× high resolution oil immersion objective and bright field microscope optics (Köhler illumination).

**Sperm morphology assessment**
☐ Tygerberg Strict Criteria were used for the evaluation of human sperm morphology. Note: Another classification could be used for scientific studies with specific aims if the classification is described or referenced. Depending on the aim of the study, the evaluation of particular abnormal forms might be useful.
NB: The classification used is the modified David classification described by Auger et al. in 2016 [47].
☐ Abnormalities are recorded for all four regions of the spermatozoon (head, neck/midpiece, tail and cytoplasmic residue) and the Teratozoospermia Index or 'TZI' was calculated.
NB: Not applicable. The classification used is the modified David classification, which allows the calculation of the multiple anomalies index (MAI).
☐ If the laboratory claims to use Tygerberg Strict Criteria for the evaluation of human sperm morphology, then the laboratory must participate in an external quality assurance scheme to verify that its assessments comply with these criteria.
NB: Not applicable. The classification used is the modified David classification. However, our laboratory participated in an external quality assurance scheme.
☐ The Papanicolaou staining method adapted for the assessment of human sperm morphology was used. For specific aims other staining methods could be used, but must then be declared and explained.
NB: Our laboratory uses Shorr staining methods, which is the method used for the modified David classification [47].
☒ At least 200 spermatozoa were assessed in each ejaculate.

☒ Assessments were done under high magnification (×1000– 1250) using a 100× high resolution oil immersion objective and bright field microscope optics (Köhler illumination).

**Other findings**
☒ The presence of abnormal clumping (aggregates and agglutinates) was recorded.
☒ Abnormal viscosity was recorded.

☒ The presence of inflammatory cells was recorded and reported if more than 1 million/mL.

☒ For the purpose of classifying infertility status (World Health Organization, 2010), antisperm antibodies were examined with a validated screening test (state which method was used: mixed antoglobulin reaction (MAR) test)

**Analysing data**

☒ The actual duration of sexual abstinence (in 'hours' or 'days') was recorded for each sample and included in the data reported in the manuscript.

☒ As a minimum in clinical studies, semen volume, sperm concentration, total number of spermatozoa/ejaculate, and abstinence time are given to reflect sperm production and output; only samples identified as having been collected completely can be included in the study.

☐ Confounding factors have been considered for statistical analysis: e.g., abstinence time and age, to evidence secular or geographical variations in sperm concentration or sperm count.

NB: The sperm characteristics presented in this study were used only to classify patients, and they were not used here as variables affecting our results.

☐ If appropriate, optional biochemical markers for prostatic, seminal vesicular and epididymal secretions were analysed and reported both as concentration and total amount.

NB: This was not necessary for this study.
